# *XRDlicious*: an interactive web-based platform for online calculation of diffraction patterns and radial distribution functions from crystal structures

**DOI:** 10.1107/S1600576725005370

**Published:** 2025-08-08

**Authors:** Miroslav Lebeda, Jan Drahokoupil, Petr Veřtát, Šimon Svoboda, Vojtěch Smola, Ubaid Ahmed, Petr Vlčák

**Affiliations:** a Faculty of Mechanical Engineering Czech Technical University in Prague Technická 4 Prague 6 16607 Czechia; b Faculty of Nuclear Sciences and Physical Engineering Czech Technical University in Prague Trojanova 339/13 Prague 2 12000 Czechia; c FZU – Institute of Physics of the Czech Academy of Sciences Na Slovance 2 Prague 8 18200 Czechia; Goa University, India

**Keywords:** X-ray diffraction, neutron diffraction, radial distribution functions, diffraction pattern simulators, web-based tools, powder diffraction data conversion

## Abstract

*XRDlicious* is an online computational tool for calculating powder X-ray diffraction and neutron diffraction patterns (diffractograms) and partial and total radial distribution functions [(P)RDFs] from crystal structures. It features a user-friendly browser-based interface that supports widely used structural file formats, such as CIF, POSCAR, XYZ and LMP. The structures can be either uploaded or retrieved directly from the Crystallography Open Database, Materials Project and Automatic FLOW databases via an integrated search interface. Uploaded structures can be modified and exported in CIF, POSCAR, LMP or XYZ formats. The tool also enables the conversion of experimental powder diffraction data between different wavelengths, to *d*-space or *q*-space representations, and between fixed or automatic divergence slits. By eliminating the need for software installation, this application ensures accessibility across various devices (including mobile phones and tablets) and different computer operating systems. Its ability to simultaneously plot diffraction patterns from multiple structures is particularly useful for comparing structures obtained from computational simulations. Beyond research applications, its intuitive interface also makes it suitable for educational purposes, allowing students to explore concepts such as diffraction patterns and (P)RDFs in an easily approachable way. *XRDlicious* is accessible at https://xrdlicious.com.

## Abstract 2

*XRDlicious* is an online tool for calculating powder X-ray and neutron diffraction patterns, as well as (partial) radial distribution functions, from crystal structures uploaded by users or retrieved from major structural databases. Its browser-based interface allows structure modification, data format conversion and simultaneous plotting for comparison, making it accessible for both research and educational purposes without the need for software installation.

## 1. Introduction

Powder X-ray diffraction (XRD), neutron diffraction (ND), and the analysis of partial and total radial distribution functions [(P)RDFs] are essential techniques for the structural characterization of materials. The interpretation and simulation of data from these techniques rely on computational tools capable of generating diffraction patterns (diffractograms) and distribution functions from known structural information. While a variety of sophisticated computational tools are available for these calculations [*e.g.Atomsk* (Hirel, 2015 ▸), *VESTA* (Momma & Izumi, 2011 ▸), *FullProf Suite* (Rodríguez-Carvajal, 1993 ▸), *TOPAS* (Coelho, 2018 ▸), *PowderCell* (Kraus & Nolze, 1996 ▸), *JANA2006*/*JANA2020* (Petříček *et al.*, 2014 ▸), *XRDplayground* (Estrada & Malfatti-Gasperini, 2025 ▸), *CrysX* (Sharma & Mishra, 2019 ▸), or Python libraries such as *Atomic Simulation Environment* (*ASE*) (Larsen *et al.*, 2017 ▸), *Pymatgen* (Ong *et al.*, 2013 ▸) and *Matminer* (Ward *et al.*, 2018 ▸)], many impose limitations by requiring local installation, licensing, scripting or operating system compatibility. Such constraints impede accessibility, especially for researchers seeking quick assessments on diverse platforms, including mobile devices and tablets. Given these limitations, online web-based tools present a practical and accessible alternative, usable across different devices and operating systems without the need for installation.

At present, the only established web-based online tools for computing powder diffraction patterns directly from crystal structures are the Inorganic Crystal Structure Database (ICSD) (Belsky *et al.*, 2002 ▸) and the Materials Project (MP) (Jain *et al.*, 2013 ▸) [to the best of our knowledge, also supported by commonly used search engines such as Google and emerging AI-powered platforms like Perplexity (Li & Sinnamon, 2024 ▸)]. However, access to the ICSD requires a license, and users are limited to structures available in its database, with no option to upload their own files. The MP is freely accessible upon registration and offers a wide range of functionalities, also including the ability to calculate powder XRD patterns for structures within its database. Although it allows users to upload their own structures, the direct calculation of XRD patterns for these uploaded structures appears to be unavailable, thus limiting its XRD functionality to its pre-existing database entries. Moreover, the platform’s broad scope can make finding specific functionalities challenging, and some users may therefore remain unaware of the available XRD computation feature.

These factors limit the practical accessibility of simulation tools within both the ICSD and MP, especially for researchers who need to compute diffraction patterns from user-provided structural data files, such as those representing atomic configurations obtained from *ab initio*, molecular dynamics or Monte Carlo simulations. This capability is relevant, for example, when comparing diffraction patterns or (P)RDFs of structures before and after geometry optimization. Moreover, these limitations extend to educational purposes, where students or users lacking deep expertise in diffraction would benefit from hands-on opportunities to simulate diffraction patterns and (P)RDFs using their own structural data within an accessible web-based environment.

In response to these needs, we introduce a web-based online calculator called *XRDlicious*, featuring a user-friendly interface designed to compute powder diffraction patterns and (P)RDFs directly from multiple crystal structure files (accessible at https://xrdlicious.com). This tool provides an intuitive browser-based interface that accepts standard crystallographic and computational structure file formats (*e.g.* CIF, POSCAR, XYZ and LMP), enables real-time graphical visualization of structures and plots (graphs), and facilitates straightforward data export.

To streamline the acquisition of structure files, *XRDlicious* includes an integrated search interface for retrieving structures from databases such as the MP, Automatic FLOW (AFLOW) (Curtarolo *et al.*, 2012 ▸; Esters *et al.*, 2023 ▸) and Crystallography Open Database (COD) (Gražulis *et al.*, 2009 ▸). Additionally, it enables conversion between primitive and conventional unit-cell representations, and modification of elemental composition, atomic occupancies and lattice parameters, with the option to download the corresponding modified structure files in CIF, POSCAR, LMP or XYZ formats (suitable for computer simulations as a convertor between different file formats). In addition to structural data handling, *XRDlicious* supports the conversion of experimental powder diffraction data between different wavelengths, to *d*-space and *q*-space representations, and between fixed and automatic divergence slit geometries.

By eliminating the need for software installation, licensing or registration, our approach broadens access to crystallographic insights. Furthermore, its ease of use makes it suitable for educational purposes, providing students and those for whom diffraction is not a primary focus an accessible platform to explore structural properties and diffraction behavior. This article describes the tool’s implementation, operational guidelines and functional capabilities, illustrating its potential applications in crystallography and materials research.

## 2. Program description

### 2.1. Program implementation

*XRDlicious* has been developed in Python using the *Streamlit* library (https://streamlit.io/) to create an interactive web-based user interface. We distribute it under the MIT license. An overview of the packages used in *XRDlicious*, along with their categorization, is provided in Table 1 ▸. *NumPy* (https://numpy.org/) is used for numerical operations and *Plotly* (https://plotly.com/) for plots and structure visualization together with *Py3Dmol* (Rego & Koes, 2015 ▸). Uploaded crystal structure data files are processed by *ASE* (Larsen *et al.*, 2017 ▸) or *Pymatgen* (Ong *et al.*, 2013 ▸). The *Matminer* (Ward *et al.*, 2018 ▸) library is utilized for the computation of (P)RDFs and the *Pymatgen* library (https://pymatgen.org/) performs diffraction pattern calculations. The *Pillow* library (https://pypi.org/project/pillow/) is employed for using illustrative images within the application. All libraries used in this application are distributed under free and open-source licenses.

Access to information within the MP, AFLOW and COD databases is achieved through their respective, freely accessible application programming interfaces (APIs). Specifically, data retrieval utilizes the Python packages provided by MP and AFLOW for their APIs, while a RESTful API is employed for interaction with the COD. For online accessibility, *XRDlicious* is currently deployed on the Streamlit Community Cloud, a free cloud-based service. The source code is publicly available on GitHub (https://github.com/bracerino/xrdlicious), where users can also find instructions for downloading and running the application locally.

### 2.2. Data input and computational module selection

**Figure 1:**
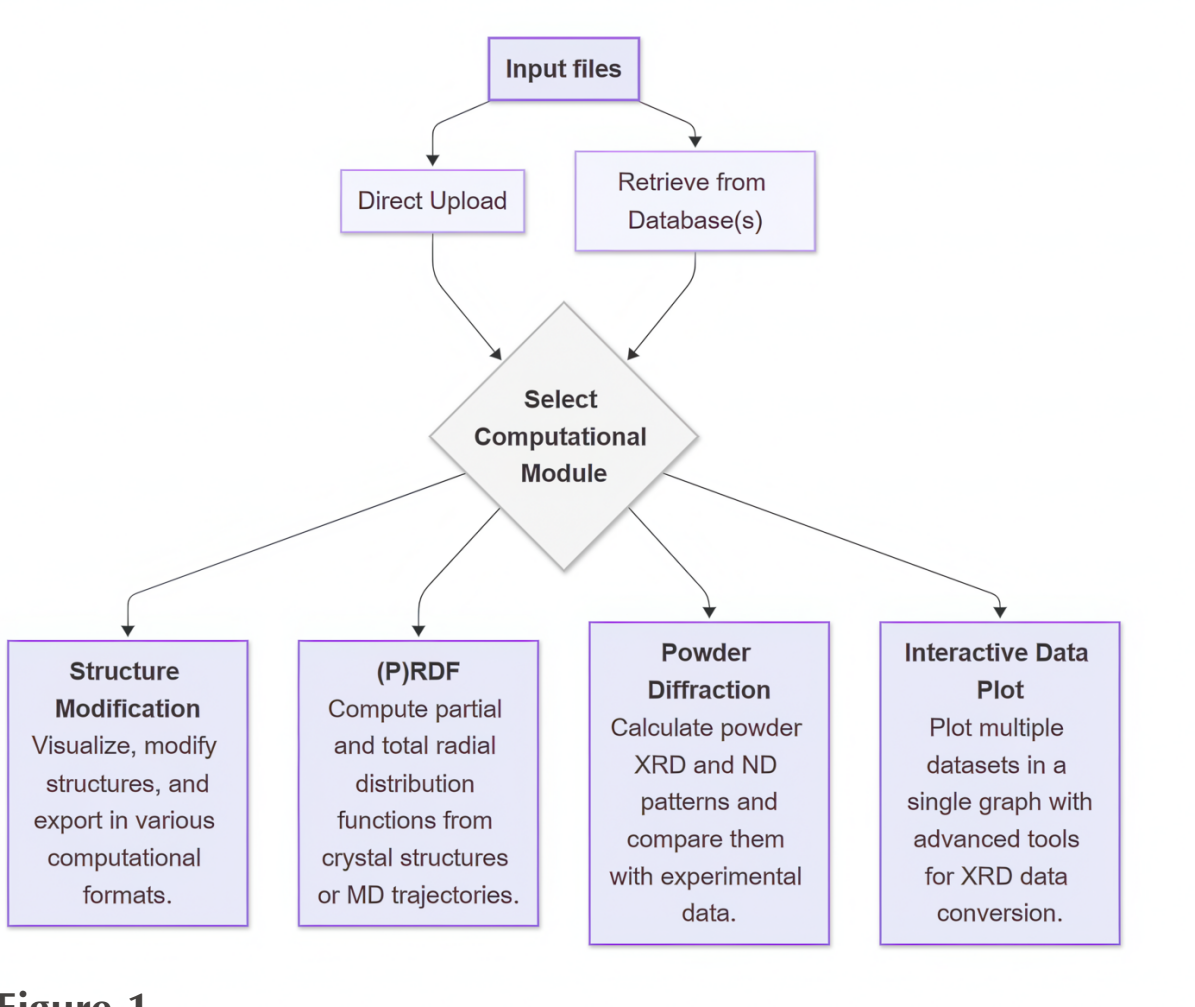
A block diagram illustrating the workflow of *XRDlicious* with currently available computational modules.

A brief overview and video tutorials for using the *XRDlicious* application are available at https://implant.fs.cvut.cz/xrdlicious/. The *XRDlicious* workflow begins with data input, as illustrated in the block diagram in Fig. 1 ▸. Users must initially provide their input files through one of two methods. The first method includes direct upload of local files. The platform supports a wide range of crystal structure formats (see Table 2 ▸), including CIF (crystallographic information file), POSCAR (*VASP*; Kresse & Furthmüller, 1996 ▸), LMP or DATA (*LAMMPS*; Thompson *et al.*, 2022 ▸), XYZ (with lattice information), XSF (*XCrySDen*; Kokalj, 1999 ▸), PW (*Quantum ESPRESSO*; Giannozzi *et al.*, 2009 ▸) and CFG (*QSTEM*; Koch, 2002 ▸). For experimental data, the software accepts two-column text files with common delimiters (space, tab, colon or semicolon). The second method allows for crystal structures to be imported through an integrated search interface, connected to the MP, AFLOW and COD databases.

**Figure 2:**
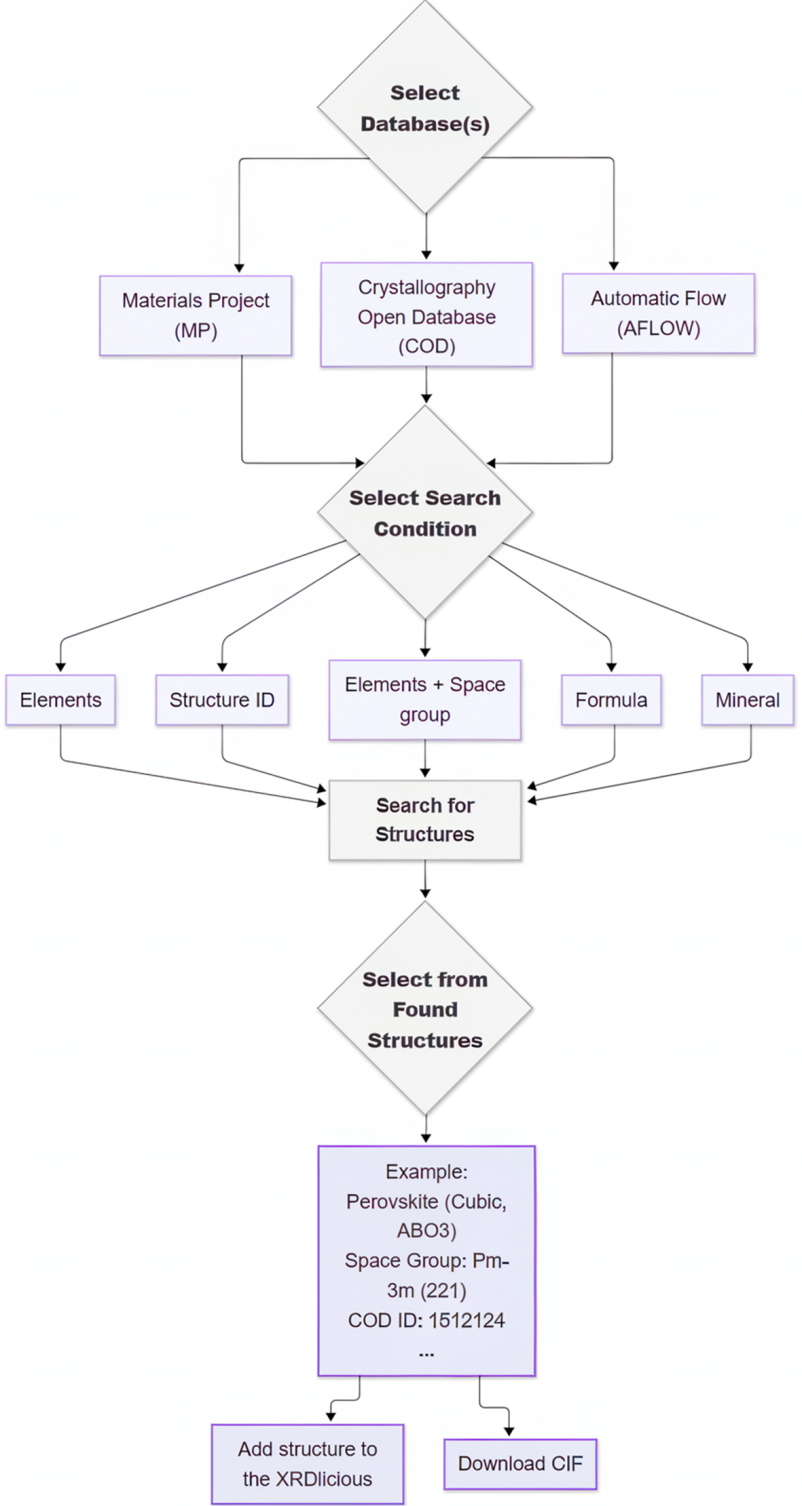
A block diagram illustrating the process of structure retrieval using the implemented search interface for crystal databases.

Within the database search interface, users can currently restrict the search by defining the set of databases (MP, AFLOW, COD) and applying one of the following criteria (see also the block diagram in Fig. 2 ▸):

(1) Allowed elements (*e.g.* Sr Ti O).

(2) Structure IDs (*e.g.* mp-5229, cod_1512124, aflow:010158cb2b41a1a5).

(3) Allowed elements + space-group number (*e.g.* Sr Ti O, 221) (the correspondence between space-group numbers and their Hermann–Mauguin symbols is also provided).

(4) Formula (*e.g.* Sr Ti O3).

(5) Mineral [*e.g.* anatase (TiO2)].

Once the search is finished, it is possible to select any found structure and either import it directly into the calculator or download it as a CIF for manual upload or other use.

After loading the input files, users can select one or more of the following computational modules. The main, currently implemented, modules include:

(1) Structure visualization and modification – allows users to visualize uploaded structure files or perform basic structure modifications. The supported features include changing atomic elements and partial occupancies, adjusting lattice parameters, and converting between primitive and conventional cells. The structures can be exported in CIF, POSCAR, LMP or XYZ formats. These export options are particularly useful for preparing input files compatible with computational packages such as *VASP* and *LAMMPS*.

(2) (P)RDF calculator – computes a (P)RDF either from the uploaded structure files or from XYZ or *LAMMPS* molecular dynamics trajectories (.lammpstrj, .dump). For the XYZ/*LAMMPS* trajectories, it is however necessary to compile the code locally due to the high computational demand. In this case, users can choose between averaging the results over all frames or computing each frame individually to create animations showing how the (P)RDF evolves with the simulation time.

(3) Powder XRD and ND calculator – calculates powder XRD and ND patterns from uploaded structures. It also enables plotting of experimental data and background subtraction for direct comparison with the computed profiles.

(4) Interactive data plot – allows users to plot multiple two-column datasets within a single interactive graph. For XRD data, the module includes advanced features such as automatic vertical stacking of multiple data files, conversion between different wavelengths, *d* spacings and *q* values, and the ability to recalculate between automatic and manual divergence slit geometries.

In the following four sections, we will describe functionalities of each of these modules in more detail.

### 2.3. Structure visualization and modification

**Figure 3:**
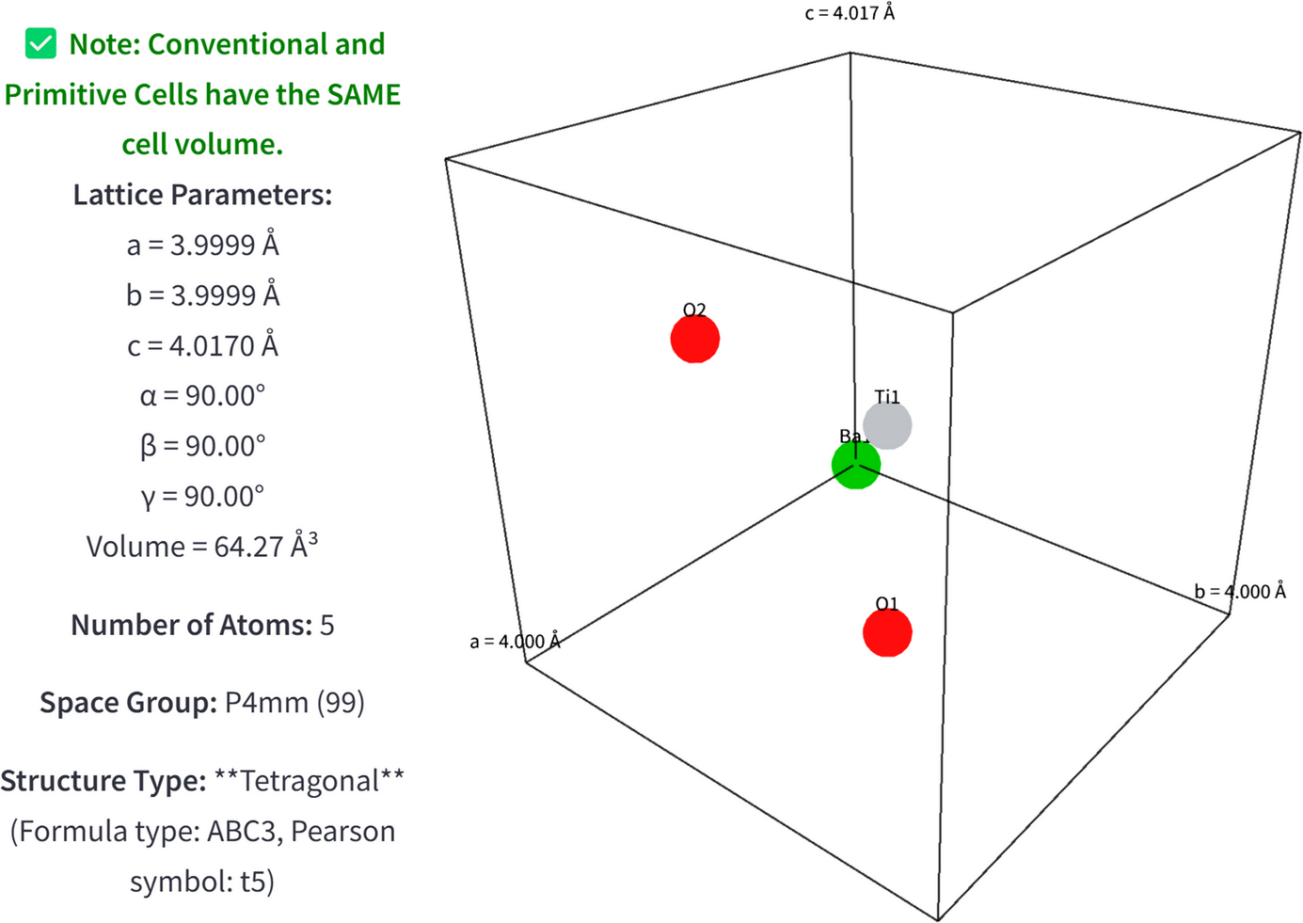
Example of an interactive structure visualization for tetragonal BaTiO_3_ (Al-Shakarchi & Mahmood, 2011 ▸), showing only atoms in the asymmetric unit.

The structure visualization module enables users to verify whether the uploaded structures correspond to their intended input (see an example in Fig. 3 ▸). Users can convert between different cell representations, including the conventional unit cell, primitive cells with Niggli (Santoro & Mighell, 1970 ▸; Shi & Li, 2022 ▸) or Lenstra–Lenstra–Lovász (LLL) (Lenstra *et al.*, 1982 ▸) lattice reduction (to minimize skewness), or a primitive cell without any further lattice reduction. Note that AFLOW only allows primitive cells without any further lattice reduction to be retrieved via its API.

Beyond visualization, the tool offers structure editing capabilities. Currently, it is possible to modify atomic elements, partial occupancies and lattice parameters. Lattice parameters can be edited while maintaining the original symmetry, or symmetry constraints can be removed to allow arbitrary lattice modifications. Additionally, the asymmetric unit can be visualized on the basis of Wyckoff positions.

Modified structures can be saved within the platform, enabling users to, for instance, compare calculated XRD patterns between the original and altered structures. The original or edited structures can be exported in a variety of common formats, including CIF, POSCAR (with option for selective dynamics), LMP (in different units and atomic styles) and XYZ. This can be practical when users wish to convert *e.g.* a CIF into a file format compatible with computational codes (POSCAR for *VASP* and LMP for *LAMMPS*).

### 2.4. (P)RDF calculations

**Figure 4:**
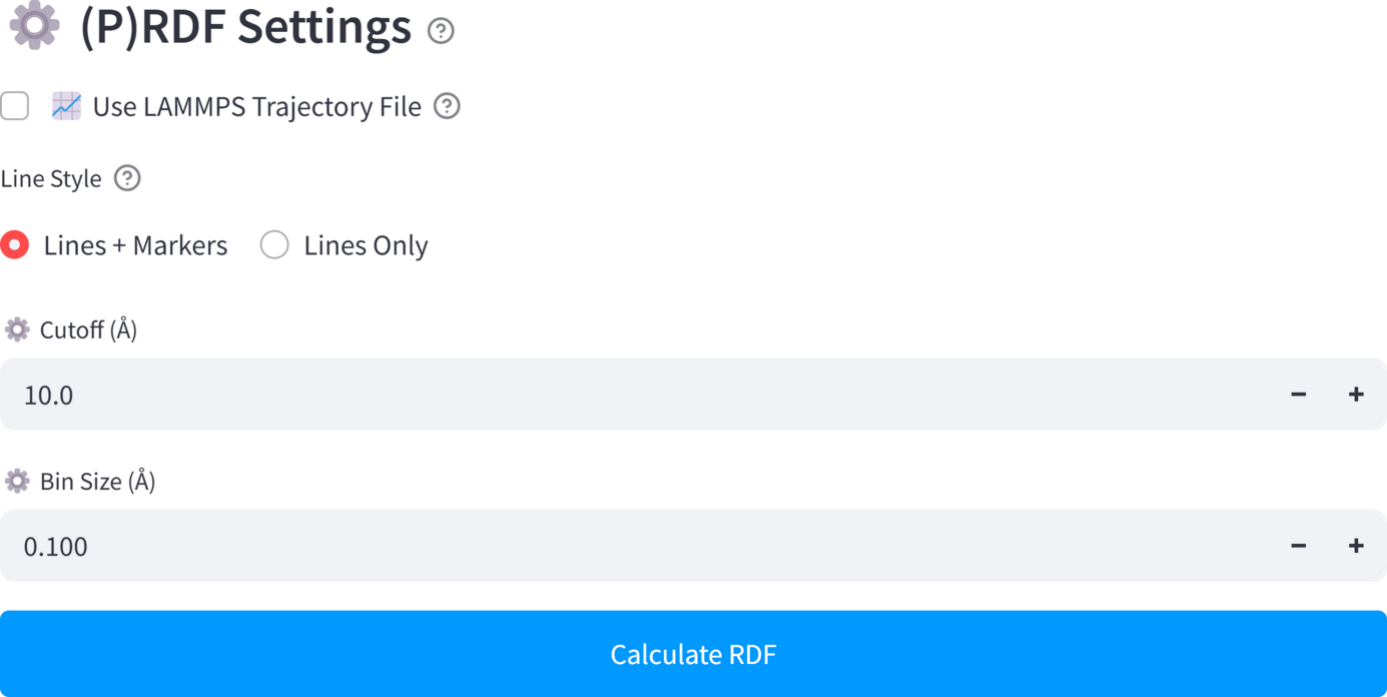
Configuration interface for (P)RDF calculations.

In the (P)RDF module, users can specify the distance cutoff (Å) and the bin size (Å) (Fig. 4 ▸). The ‘Calculate RDF’ button initiates the calculation. The software then generates PRDF plots for each element-pair combination, along with the total RDF plot. An option to download the corresponding numerical data is also available. The calculated (P)RDF values are unitless, representing relative intensities: peak positions indicate preferred bonding distances, peak widths reflect the degree of structural disorder, and peak heights correspond to the relative likelihood of those distances.

**Figure 5:**
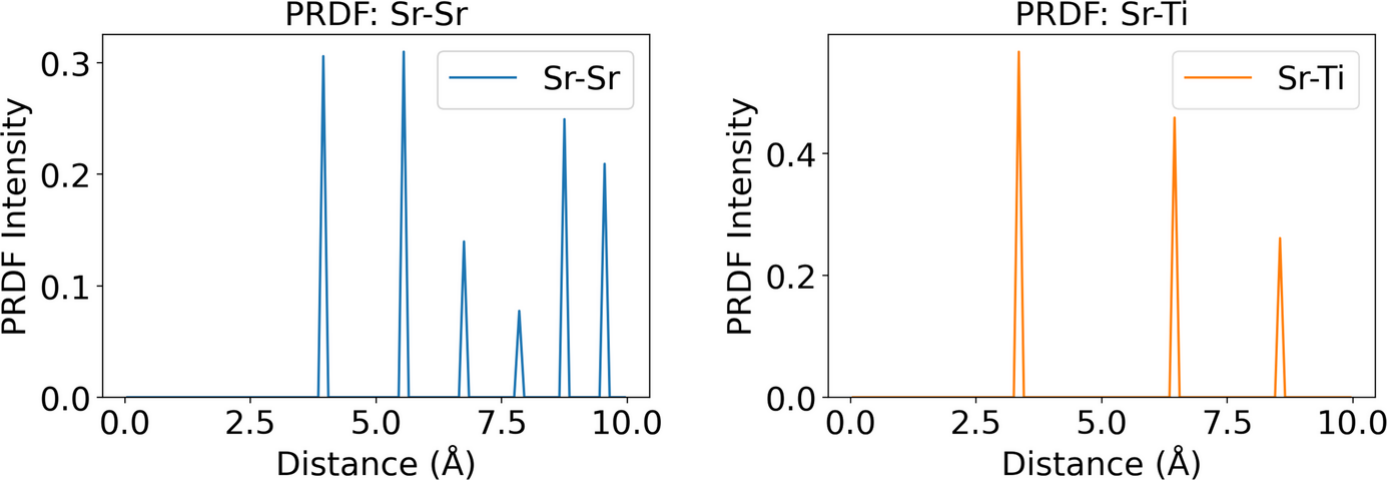
An example of the computed (P)RDF for the cubic SrTiO_3_ structure. The figure displays the PRDF plot for the Sr–Sr and Sr–Ti element pairs.

An example of the computed (P)RDF for cubic SrTiO_3_ (Longo *et al.*, 2010 ▸) is given in Fig. 5 ▸, which shows the PRDF plot for the Sr–Sr and Sr–Ti element pairs.

In addition to structure files, users can upload *LAMMPS* or XYZ trajectory files. However, this option is only available when the application is compiled and run locally due to the potentially high computational demands [trajectory files can span hundreds of megabytes, and the calculation of the (P)RDF for each frame may require significant memory resources]. Two analysis modes are provided for trajectory data: (i) calculation of the average (P)RDF across all frames, and (ii) calculation of the (P)RDF for each individual frame. The latter option generates a plot with an animation feature to visualize how the (P)RDF evolves over the course of the simulation.

### 2.5. Powder XRD and ND diffraction pattern calculations

The powder diffraction patterns are computed under the assumption of randomly oriented crystallites. For XRD, the calculation includes the Lorentz–polarization (Lp) correction, defined as Lp(θ) = [1 + cos^2^(2θ)]/(sin^2^ θ cos θ). It does not currently account for additional corrections such as preferred orientation, absorption and instrumental broadening. The same applies to the ND calculation, with the main differences from XRD being that only the Lorentz factor is applied [L(θ) = 1/(sin^2^ θ cos θ)], as the polarization factor is not needed, and constant atomic scattering lengths are used. The peak shapes can be modeled either as a delta function or using a Gaussian function, characterized by a sigma parameter. Temperature effects can be considered by defining Debye–Waller factors for each element in each structure file.

**Figure 6:**
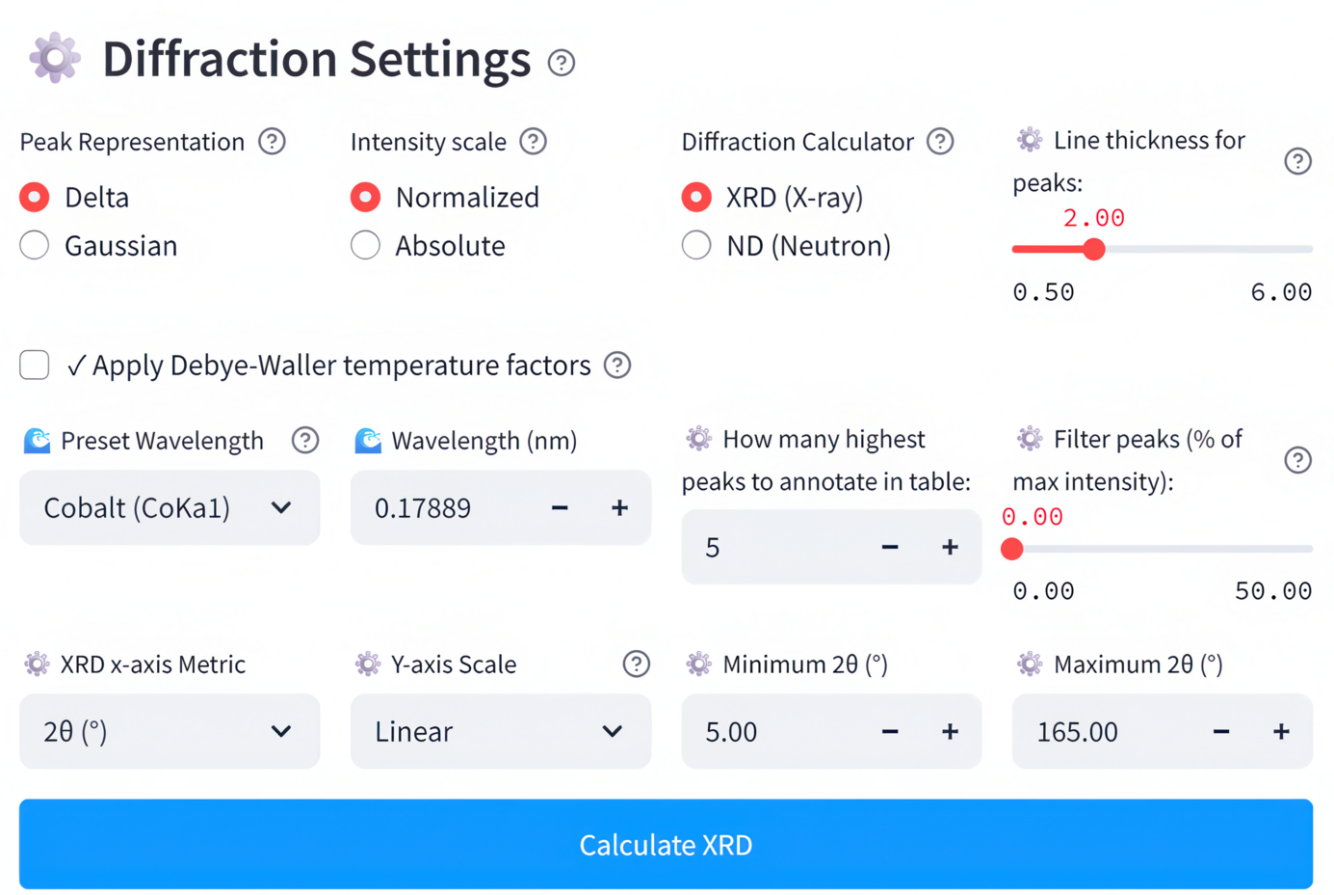
Configuration interface for the powder diffraction pattern calculation.

Regarding the powder diffraction pattern calculation settings (Fig. 6 ▸), users can select X-rays or neutrons and choose from predefined X-ray wavelengths corresponding to different radiation sources [Cu, Mo, Co, Cr, Fe, Ag with *K*α_1_, *K*α_2_, *K*β_1_, (*K*α_1_ + *K*α_2_), (*K*α_1_ + *K*α_2_ + *K*β_1_)] or neutron wavelengths corresponding to thermal, hot or cold neutrons. Alternatively, users may manually enter a custom wavelength value. The *X*-axis metric for the diffraction pattern can be chosen from 2θ (°), 2θ (rad), θ (°), θ (rad), *q* (Å^−1^), *q* (nm^−1^), *d* (Å), *d* (nm), *E* (keV), *f* (PHz). Additionally, users can specify minimum and maximum *X*-axis values to focus on a specific range and adjust the Gaussian sigma parameter to control peak broadening. Finally, the number of the most intense peaks to annotate can be specified, and there are options to display intensities either as normalized values or in absolute units and omit peaks with intensity lower than a configurable threshold.

Clicking ‘Calculate XRD/ND’ generates the diffraction patterns and the corresponding data, which include peak position, intensity and (*hkl*) indices. The output consists of two expandable sections: one containing the complete list of peaks and another showing only the most intense peaks based on the selected number for annotation. Once the diffraction pattern is generated, any parameter changes automatically trigger recalculation and real-time plot updates.

**Figure 7:**
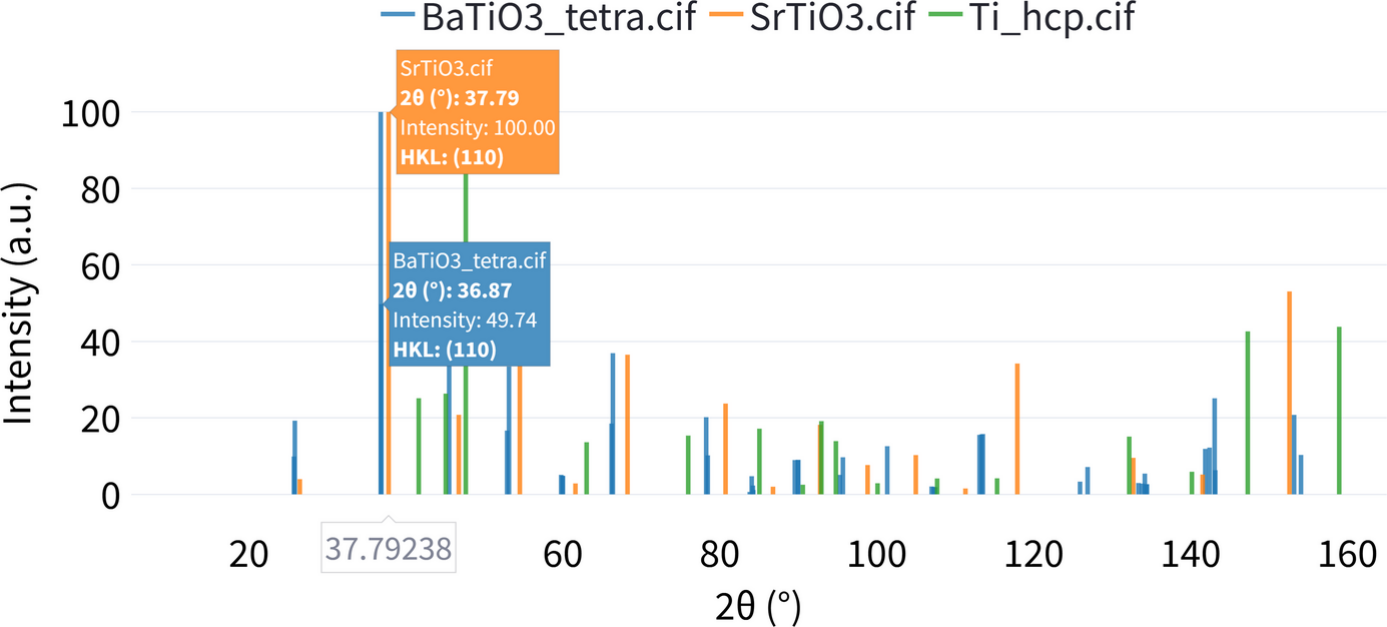
Interactive plot of the computed powder XRD patterns for three example structures.

**Figure 8:**
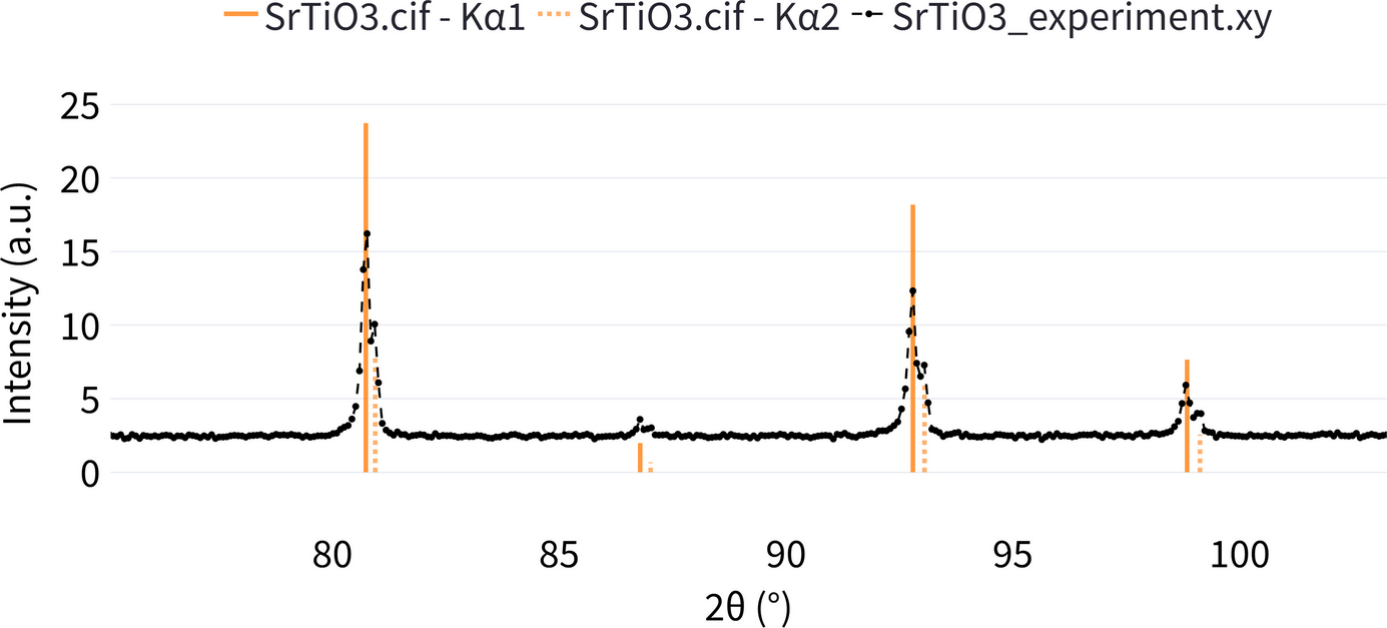
Comparison between uploaded experimental XRD data and the simulated XRD pattern for the SrTiO_3_ example.

The computed powder XRD patterns for example structures of cubic SrTiO_3_, tetragonal BaTiO_3_ and hexagonal α-Ti (Novoselova *et al.*, 2004 ▸) are displayed in Fig. 7 ▸. The output provides an interactive plot where users can hover over the peaks and see their corresponding phase (file name), *x*-axis position, intensity and (*hkl*) indices. Furthermore, an interactive table is presented, containing the following columns: *X*-axis value (according to the selected metric), intensity, (*hkl*) indices and phase (structure name). Additionally, users can upload their own experimental diffraction data for comparison with the computed patterns (Fig. 8 ▸).

### 2.6. Interactive data plot and data conversion

**Figure 9:**
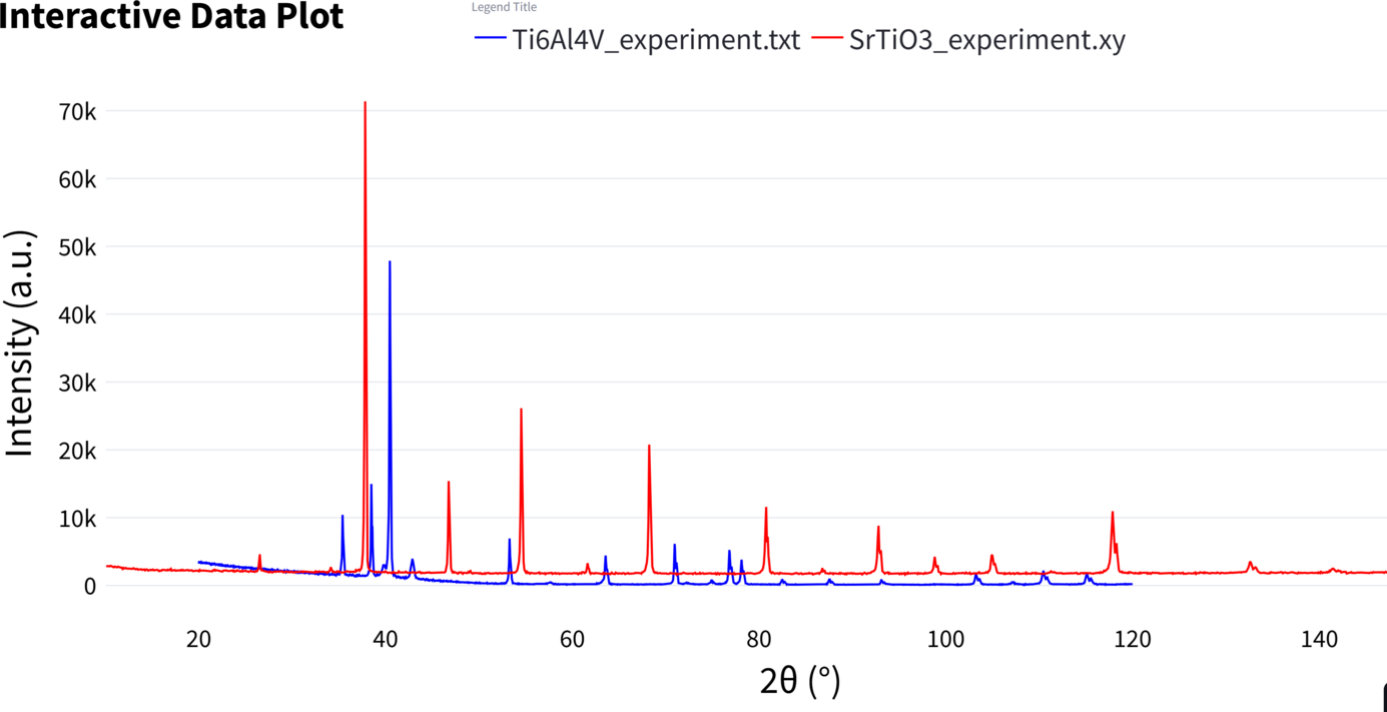
Example of a plot generated in the ‘Interactive Data Plot’ module, showing two experimental datasets.

The ‘Interactive Data Plot’ tool allows users to upload and visualize multiple two-column datasets in an interactive plotting interface (see Fig. 9 ▸ for an example featuring two uploaded experimental datasets). Users can customize the plot layout and apply transformations to the axes, such as converting the *x* and *y* axes to logarithmic scales. Additionally, the tool enables vertical scaling of individual datasets through offsetting or multiplication. It is also possible to automatically find the offsets between more data files and stack them vertically.

For XRD datasets, an additional feature enables data conversions. This includes the ability to convert between different X-ray wavelengths or to transform the *x* axis into either *d* spacing (Å) or scattering vector magnitude *q* (Å^−1^) representations.

**Figure 10:**
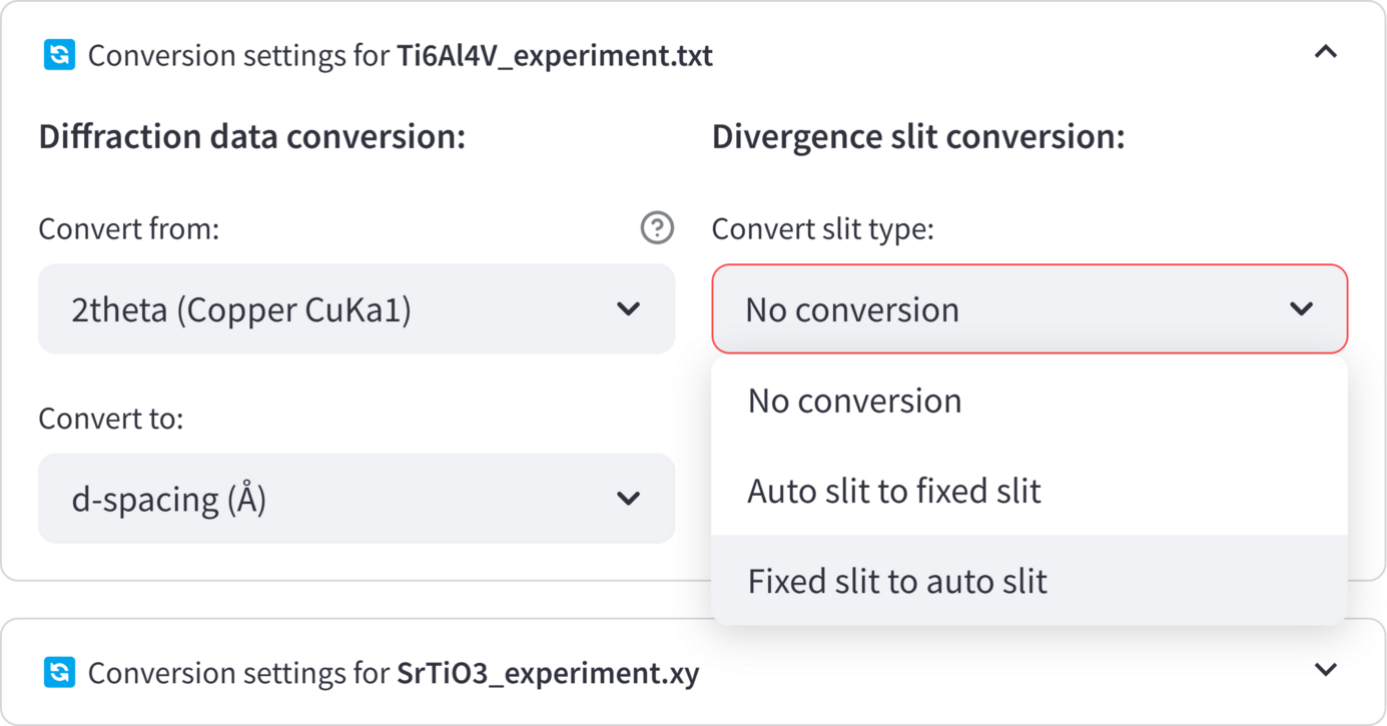
Configuration interface for the powder XRD data conversion.

The tool also provides conversions between diffraction patterns acquired using automatic and fixed divergence slit configurations (see Fig. 10 ▸ for the configuration interface). The intensity transformations between fixed (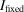) and automatic (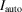) divergence slits are based on the following equation:
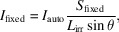
where 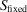 is the fixed slit size (angular width) and 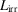 is the irradiated sample length. These two parameters can be adjusted according to the user requirements.

### 2.7. Limitations

While our tool enhances accessibility for calculating powder XRD/ND patterns and (P)RDFs, it possesses certain limitations. Currently, the software’s ability to read structural data relies on *ASE* and *Pymatgen* supported formats, which include several common file types. However, formats specific to certain simulation software might result in errors, for instance, *VESTA* (.vesta), *ABINIT* (Gonze *et al.*, 2020 ▸) (.in) and *GULP* (Gale, 1997 ▸) (.gin). Therefore, at present, we recommend that users upload structure files primarily in the CIF, POSCAR, LMP, XYZ (with cell information included), XSF, PW or CFG formats. Additionally, due to hosting constraints associated with the *Streamlit* platform, uploading a large number of structures simultaneously might lead to slower performance, reduced responsiveness or calculation failures.

Since *XRDlicious* is currently hosted on *Streamlit*’s free, cloud-based Community Cloud, it is subject to a RAM limit of 2.6 GB. To enhance performance when working with larger datasets or structure files, users can run the application locally by cloning the repository from GitHub (see Section 2.1 for the link). Running *XRDlicious* locally removes cloud-based constraints, with performance limited only by the specifications of the user’s own system.

### 2.8. Future updates

In the future, we aim to enhance performance and further optimize the code, while also expanding the tool with new functionalities. These include calculating the effect of sample displacement and texture, improving the structure modification tool, introducing an option to select a peak shape function (Gaussian, Lorentzian, pseudo-Voigt), and implementing more detailed effects on peak broadening. We also plan to broaden support for additional structure file formats.

Additionally, we intend to introduce basic and advanced user modes. The basic mode will offer only the essential features for calculating diffraction patterns, making it even more accessible to students or users unfamiliar with diffraction techniques. This should help streamline the user interface and avoid potentially overwhelming complexity. The advanced mode, on the other hand, will provide expanded functionalities for more experienced users. *XRDlicious* will be continuously updated and improved to reflect user feedback and research demands.

## 3. Conclusion

In this work, we have introduced *XRDlicious*, a newly developed user-friendly web-based calculator for computing powder XRD or ND patterns, PRDFs and total RDFs from multiple crystal structure files. It supports a variety of widely used formats, including CIF, POSCAR, LMP and XYZ, and enables direct inclusion of structures from the MP, AFLOW and COD databases via an integrated search interface. Our application allows users to configure main calculation parameters for both (P)RDFs and diffraction patterns, visualize results, and export data for further analysis. *XRDlicious* also supports basic structural modifications and the conversion of experimental data across different wavelengths or to *d*-space or *q*-space representations.

The tool runs online directly in a browser, ensuring compatibility across various devices (mobile phones, tablets, computers). Its capability to simultaneously plot patterns for multiple structures makes it especially suitable for comparative analyses of structural variations, for example, those resulting from computer simulations. In addition to its utility in research, the user-friendly interface makes *XRDlicious* well suited for students and users with limited diffraction expertise, providing hands-on experience in diffraction and structural characterization.

## Figures and Tables

**Table 1:** Categorization of used Python packages in *XRDlicious*

Web interface	*Streamlit*, *Streamlit*–*Plotly* events
Plotting/visualization	*Matplotlib*, *Plotly*, *Py3Dmol*, *Pillow*
Numerical operations	*NumPy*
Materials science libraries	*ASE*, *Matminer*, *Pymatgen*, *pymatgen.analysis.defects*, *mp-api*, *AFLOW*

**Table 2:** Supported formats for crystal structures and experimental data files

Type	Format	Description
Crystal structure file	.cif	Crystallographic information file
	POSCAR	*VASP* structure file
	.lmp, .data	*LAMMPS* structure file
	.xyz	XYZ format (must include cell information)
	.xsf	*XCrySDen* structure file
	.pw	*Quantum ESPRESSO* file
	.cfg	*QSTEM* configuration file
Experimental data file	.xy, .csv, .dat, .data, .txt	Two-column text files separated by space, tab, colon or semicolon
